# Study of prompt gamma and neutron emission for real‐time range verification in proton and carbon‐ion therapy

**DOI:** 10.1002/mp.70536

**Published:** 2026-06-25

**Authors:** Emma Sofia Bellotti, Davide Mazzucconi, Davide Bortot, Andrea Pola, Carlo Fiorini, Korbinian Urban, Stefano Agosteo

**Affiliations:** ^1^ Department of Energy Politecnico di Milano Milano Italy; ^2^ Department of Electronics, Information and Bioengineering (DEIB) Politecnico di Milano Milano Italy

**Keywords:** hadrontherapy, prompt gamma imaging, real‐time range verification

## Abstract

**Background:**

Accurate range verification is crucial in hadrontherapy to fully exploit the ballistic advantages of charged particles and prevent damage to healthy tissues. Among the proposed approaches, prompt gamma imaging (PGI) has emerged as an effective technique for real‐time monitoring, but its performance is limited by the intense neutron background generated during irradiation, especially with carbon‐ions.

**Purpose:**

This work presents a Monte Carlo study performed with the FLUKA code to investigate prompt gamma and neutron emission in proton and carbon‐ion therapy. A prototype detection system based on a knife‐edge collimator coupled to a pixelated LYSO scintillator was simulated to evaluate its capability for range verification. The aim is to quantify how neutron fields and neutron induced signals bias or degrade range related quantities, and how these effects differ between proton and carbon‐ion beams.

**Methods:**

The analysis includes the characterization of prompt gamma energy spectra and spatial profiles, the assessment of neutron fields within a treatment room, and the decomposition of the detector signal into primary gammas, secondary gammas, and neutrons.

**Results:**

Results show that prompt gamma profiles correlate well with the Bragg peak position, particularly within the 3–7 MeV energy window, while carbon ions exhibit higher prompt gamma yields but also significantly stronger neutron backgrounds compared to protons. Detector simulations highlight the impact of neutron capture on lutetium, producing distinct peaks that must be accounted for in the detector signal analysis. The fall‐off retrieval precision (FRP) analysis indicates that the distal fall‐off of prompt gamma profiles can be used to estimate the Bragg peak position, while secondary radiation components introduce additional fluctuations that affect the achievable precision, particularly for carbon‐ion beams.

**Conclusions:**

The study provides a detailed characterization of prompt gamma and neutron contributions in proton and carbon‐ion therapy and highlights the main physical factors affecting PGI‐based range monitoring, particularly in the presence of neutron‐induced backgrounds. These results provide useful insights for the design and optimization of prompt gamma detection systems in clinical applications.

## 1 INTRODUCTION

Hadrontherapy is an advanced radiotherapy technique that allows for the effective treatment of deep‐seated and radio‐resistant tumors, as well as tumors located near critical organs. These advantages are related to the use of charged particles, such as protons and heavier ions (i.e., carbon‐ions), to deliver the dose with higher spatial precision compared to conventional photon therapy.^1^, ^2^ This feature is due to the physical characteristics of charged particles, which deposit most of their energy at the end of their path, resulting in the so called Bragg Peak (BP). This confined distribution allows to give less dose to the surrounding healthy tissues. Additionally, carbon‐ions exhibit higher linear energy transfer (LET) and relative biological effectiveness (RBE), offering further benefits for radio‐resistant tumors.^3^

However, to fully exploit these advantages, it is essential to ensure accurate in vivo range verification, as even small uncertainties in beam range can result in underdosing the tumor or, more critically, overdosing adjacent healthy tissues. These range uncertainties arise from several sources, including limitations in CT imaging, inaccuracies in the calibration from Hounsfield units (HU) to stopping power ratios (SPR),^4^, ^5^, ^6^, ^7^ patient setup errors, anatomical changes during the treatment course, and organ motion.^8^ Additional contributions may arise from dose calculation approximations and beam delivery imperfections.^9^ To mitigate these uncertainties, clinical protocols typically incorporate both range‐dependent and absolute safety margins during treatment planning.^10^ Although this strategy increases robustness, it may partially compromise the spatial conformity of ion therapy by expanding the high‐dose region into surrounding healthy tissues.^11^

To mitigate these uncertainties, several in vivo range verification techniques have been developed; these are based on detecting secondary radiation emitted during beam irradiation, which is correlated with the beam path and dose deposition. One of the most promising methods is positron emission tomography (PET) imaging, which relies on the detection of $\beta^{+}$ emitters produced along the beam path. PET systems can be implemented in off‐line, in‐room, or in‐beam configurations. While in‐beam PET reduces the impact of biological washout, PET‐based techniques still suffer from a progressive reduction in tracer activity and from practical limitations related to background radiation and acquisition constraints, which restrict their real‐time applicability.^12^, ^13^, ^14^, ^15^ Alternative approaches include ionoacoustic and bremsstrahlung‐based methods, which determine the BP position from acoustic signals or secondary photons generated during irradiation.^16^, ^17^ Another promising technique is Interaction Vertex Imaging (IVI), which tracks secondary charged particles emerging from nuclear interactions to reconstruct their origin and estimate the beam range within the patient.^4^ Finally, it has been proposed that a small percentage of helium ions could be added to a carbon‐ion treatment beam for online treatment monitoring.

Other approaches are based on prompt gamma (PG) techniques, which exploit the almost instantaneous emission of nuclear de‐excitation photons directly correlated with the beam interaction positions. prompt gamma Spectroscopy (PGS) focuses on measuring the energy spectra of these photons to estimate the beam range with high specificity although it requires fast and low‐noise detection systems.^18^, ^19^, ^20^, ^21^ prompt gamma Timing (PGT) derives range information from the time‐of‐flight distribution of prompt gammas.^22^, ^23^ Finally, prompt gamma Imaging (PGI) reconstructs the spatial origin of prompt gammas using collimated or Compton‐based detectors, providing a visual representation of the beam path and enabling real‐time range verification.^24^, ^25^, ^26^

PGI, in particular, has emerged as one of the most effective approaches for real‐time monitoring of beam range and dose deposition.^11^, ^18^, ^19^, ^20^ Since its initial proposal in 2003,^27^ it has evolved significantly thanks to advances in detection technologies. Systems based on slit and knife‐edge collimator have achieved one and two dimensional imaging with clinical accuracy down to approximately 1–2 mm in proton therapy.^24^, ^28^, ^29^ Compton cameras, on the other hand, offer three dimensional reconstruction of gamma emission points.^25^, ^30^ More recently, time‐of‐flight (TOF) based PGI systems employing Cherenkov detectors have demonstrated millimetric sensitivity in pulsed beam conditions.^11^, ^31^

PGI systems have also been successfully translated into clinical environments, where prompt gamma imaging has been used for in‐vivo monitoring of the proton beam range during patient treatments, demonstrating the capability to detect range deviations and anatomical changes under realistic treatment conditions.^32^, ^33^, ^34^

Despite these advances, PGI continues to face significant challenges due to the intense background from neutrons and their induced photons, especially in the context of heavy ion beams, where nuclear fragmentation and the associated neutron field are significantly stronger and can dominate the measured detector signal. These limitations highlight the need for improvements in high‐resolution TOF detection,^31^, ^35^ spectral filtering,^36^ and Monte Carlo‐informed detector design.^28^, ^37^

Monte Carlo simulations are fundamental in addressing these challenges, as they enable accurate modeling of beam–matter interactions, secondary radiation generation, and detector response. Widely used codes such as fluka, geant4, and mcnp allow for the optimization of PGI system geometry, material selection, and data acquisition strategies.^28^, ^35^, ^38^, ^39^ For instance, FLUKA simulations have been used to evaluate knife‐edge collimator‐based cameras for carbon‐ion therapy, demonstrating sub‐centimetric range sensitivity even in the presence of complex radiation backgrounds.^28^ Similarly, Wang et al.^36^ used FLUKA to study a pixelated LYSO detector with shielding and energy selection, showing that spectral discrimination can effectively suppress background and improve range resolution. Finally, a recent benchmark study comparing FLUKA, GEANT4, and MCNP6 revealed discrepancies in prompt gamma yield predictions and provided guidance for cross‐code calibration.^37^, ^40^ It should be noted that, from this study FLUKA showed the more reliable results with respect to the other codes.

While most PGI studies have so far focused on proton beams,^11^ the present work extends the analysis to carbon‐ion therapy, addressing the additional challenges associated with nuclear fragmentation, neutron‐induced background and simulated detector response. Although the feasibility of prompt gamma imaging for carbon‐ions has been experimentally demonstrated, only a limited number of studies provide a systematic comparison between proton and carbon‐ion beams across clinically relevant energies within the same Monte Carlo framework.^11^, ^35^ In particular, few investigations simultaneously combine a detailed characterization of prompt gamma production in the phantom, an assessment of the neutron field in the treatment room, and a component‐resolved analysis of the detector signal, distinguishing between primary prompt gammas, neutron‐induced secondary gammas, and direct neutron contributions. For these reasons, a prototype design of a PGI system is investigated, employing a LYSO scintillation detector coupled with a knife‐edge collimator. The choice of a LYSO scintillation detector is driven by its favorable physical and technological properties for prompt gamma detection in a clinical environment. LYSO offers high density and effective atomic number, ensuring good detection efficiency in the MeV energy range, together with fast scintillation decay time and high light yield, which are essential for real‐time applications.^41^ The detector geometry investigated in this work is inspired by recently proposed LYSO‐based prompt gamma imaging prototypes employing collimated configurations,^41^ but it is enlarged with respect to existing implementations in order to allow for a detailed investigation of the spatial profiles and relative contributions of primary gammas, secondary gammas, and neutrons, allowing for a clearer interpretation of the signal. This work aims at linking the treatment room neutron field to detector effects and quantifies how each radiation component influences fall‐off–based range observables, particularly in the more challenging carbon‐ion case. In this context, the study characterizes the energy spectra and spatial distributions of prompt gammas together with background contributions from secondary gammas and neutrons, with the aim of assessing the system performance for real‐time range verification in clinical settings.

## 2 MATERIALS AND METHODS

### 2.1 Detector and simulated geometry

**FIGURE 1:**
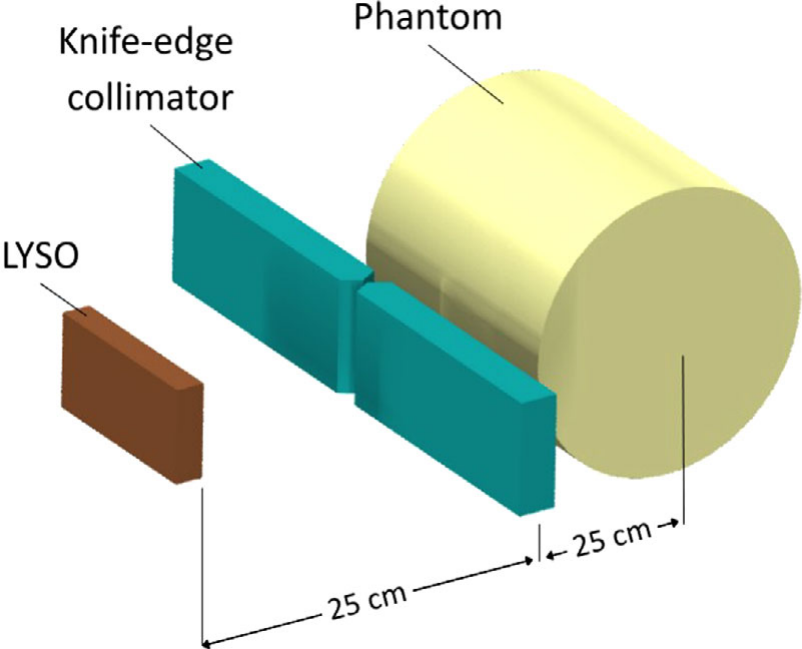
Geometry of the simulated setup implemented in FLUKA.

The simulated detection system reported in Figure 1 is based on a prototype concept currently under development. The system consists of a gamma camera, composed of a knife‐edge collimator and a pixelated scintillator detector, both aligned with the BP. The BP position is used as the reference point for the entire geometry and is always placed at the origin of the reference system, specifically at z=0 along the beam direction. Consequently, both the collimator and the detector are centered with respect to the BP and aligned with the cylindrical phantom. The collimator is placed 25 cm from the center of the phantom, while the detector is positioned 25 cm from the collimator, resulting in a total distance of 50 cm between the detector and the phantom.^28^

The collimator consists of two tungsten alloy blocks, each 15 cm long and 4 cm thick, arranged to form a 63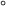 opening angle with a 6 mm gap between the knife edges. The simulated detector consist of a pixelated LYSO scintillator with an area of 20 × 10 cm^2^ and a thickness of 3 cm, segmented into 1 × 1 cm^2^ transverse pixels. For the purposes of the present analysis, the recorded signals were then grouped into 1 cm‐thick longitudinal slabs in order to extract the one‐dimensional profile along the beam axis. This choice allows for a more detailed study of the detector signal and of how the 1D Bragg peak profile, evaluated along the beam axis, is influenced by the different signal components (i.e., primary prompt gammas, neutrons, and secondary gammas).

The simulated target is a cylinder with a height of 30 cm and a radius of 15 cm, made of ICRP tissue‐equivalent material^42^ already implemented in FLUKA. Both the target geometry and material were selected to create a rather general setup, aiming to approximate, as closely as possible, the response of real tissue.

**FIGURE 2:**
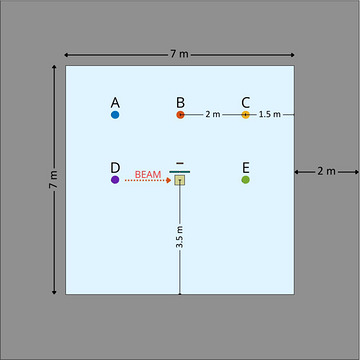
Simulated treatment room geometry and positions of the neutron fluence scoring spheres.

The entire simulated setup is positioned at the center of a room at 1.5 m above the floor. The room is 7×7 $m^{2}$ in area, 4 m high, and it is surrounded by 2 m thick walls made of Portland cement (see Figure  2). The material composition is reported in Table 1, along with the other simulated materials. This configuration was chosen to resemble an approximated hadrontherapy treatment room.^43^

**TABLE 1:** Elemental composition of the simulated materials.

Material	Mass percentages
LYSO	Lu: 71.4%, O: 18.2%, Si: 6.4%, Y: 4.0%
Tungsten alloy	W: 90%, Cu: 6%, Ni: 4%
ICRP soft tissue	O: 63.0%, C: 23.2%, H: 10.4%, N: 2.5%, others <1%
Portland cement	O: 52.9%, Si: 33.7%, Ca: 4.4%, Al: 3.4%, others <2%

### 2.2 Monte Carlo Physics

Monte Carlo simulations were performed using the FLUKA code^44^ (version 4‐4.1) with the PRECISIO setting, enabling high‐accuracy transport and interaction modelling.

Hadronic interactions are treated using different models depending on the projectile type and energy. For nucleons and pions, the PEANUT (PreEquilibrium Approach to Nuclear Thermalization) model is used up to a few GeV, accounting for intranuclear cascades, pre‐equilibrium emission, and de‐excitation processes.^45^ For ion‐induced reactions, three models are employed depending on energy: the Boltzmann Master Equation (BME) model below ∼150 MeV/u, a modified version of rQMD‐2.4 between ∼150 MeV/u and ∼5 GeV/u, and DPMJET‐III at higher energies. Electromagnetic dissociation of ions is also included via virtual photon emission coupled with the PEANUT framework.^45^ Transport thresholds in the adoptedsetting are $10^{-5}$ eV for neutrons, 10 keV for photons, and 100 keV for electrons, positrons, muons, and charged hadrons across all regions.

### 2.3 Simulation workflow and scored quantities

This study simulated two types of primary beam particles, namely protons and carbon‐ions. For each particle type, three different energies were selected, resulting in a total of six simulation sets. In particular, mono‐energetic pencil beams with energies of 60, 150, and 210 MeV for protons, and 120, 280, and 400 MeV/u for carbon‐ions, were employed. These energies were chosen to cover the energy range typically used in hadrontherapy and to obtain comparable BP depths in soft tissue^42^ for both particles: 31, 156 and 280 mm for protons, and 35, 153 and 273 mm for carbon‐ions. The Bragg Peak (BP) depth was determined from the simulated depth‐dose distributions and defined as the position of the maximum deposited dose along the beam axis. For the simulations, $10^{9}$ primary protons and $10^{8}$ primary carbon‐ions were simulated. Table 2 summarizes the beam parameters and associated Bragg peak depths used in the simulations.

**TABLE 2:** Beam energies and corresponding Bragg Peak (BP) depths in soft tissue for the simulated proton and carbon‐ion beams.^42^

Particle type	Energy	BP depth (mm)
Proton	60 MeV	31
Proton	150 MeV	156
Proton	210 MeV	280
Carbon‐ion	120 MeV/u	35
Carbon‐ion	280 MeV/u	153
Carbon‐ion	400 MeV/u	273

Various FLUKA cards and user‐defined routines were exploited for scoring data throughout the simulations. The analysis starts from the study of particles generated by the beam interaction within the phantom. Then, the neutron field produced as a consequence of neutron interactions with the room's walls and objects is analyzed. Finally, the signal scored in the simulated detector is analyzed in all its components: primary gammas, secondary gammas, and neutrons.

For the analysis of the interactions of the beam with the phantom material, an MGDRAW routine was implemented. This routine allowed the identification of the particle from which each photon originated (e.g., primary beam particles and their fragments, or other particles such as neutrons). The following classification has been adopted:

- Primary gammas: photons originated from nuclear interactions of the primary beam and its fragments with the nuclei in the phantom.
- Secondary gammas: photons produced by all the other processes, mainly those induced by secondary neutron interactions with the phantom, walls and the detector.

The MGDRAW routine allowed to save the phase space (i.e. energies, positions and directions) of photons and neutrons generated by the nuclear interactions of the primary particle with the soft tissue.

These data enabled to obtain the energy spectra and spatial profiles of the secondary radiation generated inside the phantom. Specifically, from the phase space of the primary gammas, the energy of each photon was retrieved to plot the energy spectra. The resulting spectra were binned using 10 keV intervals and are presented separately for each particle type. In addition, the spatial distribution of primary gamma production was evaluated by correlating photon energies with their production along the path of the primary particle. The profiles were computed for each beam energy with a spatial binning of 1 mm. Two approaches were applied: the first consists in analyzing the profiles using different energy selections, while the second focuses on the contribution of specific energy peaks. To further investigate the differences between beam types, the production yields of both primary gammas and neutrons were calculated.

In addition to the analysis of primary gammas, particular attention is given to the characterization of the neutron field, which represents one of the main challenges in a treatment room environment. Therefore, it is essential to characterize the neutron field generated within the treatment room during beam irradiation. To this purpose, several scoring volumes were placed at different positions in the room, and the neutron fluence (in neutrons per square centimeter) was evaluated using the FLUKA USRTRACK card.

Moreover, the signals scored in the LYSO were collected using the DETECT card, which allows for the simulation of the behavior of a detector by scoring the energy depositions inside the sensitive volume. Thanks to the information provided by the MGDRAW routine on the parent particle responsible for each interaction, the detected signal could be subdivided into different components:

- Primary gammas originating from the interaction between the beam and the phantom.
- Secondary gammas, mostly generated by neutron interactions within the room, the walls, and surrounding objects.
- Neutrons interacting directly inside the detector and depositing their energy through different processes (e.g., elastic or inelastic scattering).

The scintillator detector was subdivided into slabs, and the signal was scored independently in each of them. By integrating the counts within each slab, a one‐dimensional profile of the detector signal as a function of position along the beam axis was reconstructed. This approach enables the evaluation of the spatial distribution of each radiation field component (primary gammas, secondary gammas, and neutrons) along the detector.

### 2.4 Fall‐off retrieval precision analysis

To evaluate the statistical precision in retrieving the distal fall‐off position, a procedure following the approach proposed by *Roellinghoff et al.* ^46^ was implemented. Starting from the simulated detector profiles expressed in counts per primary particle, a reference profile was obtained by scaling the signal to different numbers of primary particles $N_{\mathrm{prim}}$. In this work, the analysis was performed for $N_{\mathrm{prim}}=10^{5},10^{6},10^{7},10^{8},10^{9}$ and $10^{10}$.

For each value of $N_{\mathrm{prim}}$, statistical fluctuations associated with counting statistics were simulated by generating 1000 noisy realizations of the signal through Poisson sampling of the expected counts in each bin. Each noisy profile was then compared with shifted versions of the reference profile. The reference curve was translated along the beam axis within a ±2 cm interval using a shift step of 0.01 cm.

The similarity between the noisy profile and each shifted reference curve was quantified using the root mean square error (RMSE). The shift corresponding to the minimum RMSE was taken as the reconstructed fall‐off displacement. Repeating this procedure for many noisy realizations yields a distribution of reconstructed fall‐off shifts. The statistical uncertainty in the fall‐off determination is quantified from the width of this distribution, and the fall‐off retrieval precision (FRP) is defined as twice the standard deviation of the reconstructed shift distribution, corresponding to a 2σ confidence interval.

## 3 RESULTS

### 3.1 Radiation from Beam–Phantom interactions

At this stage, only the interactions between the beam particles and the phantom are presented; therefore, all the contributions coming from the room and the other objects are neglected. It should be noted that only the first photons generated by hadronic particle interactions were scored: photons resulting from the following interactions of primary photons or neutrons were not included in this initial analysis.

#### 3.1.1 Energy spectra and depth profiles

**FIGURE 3:**
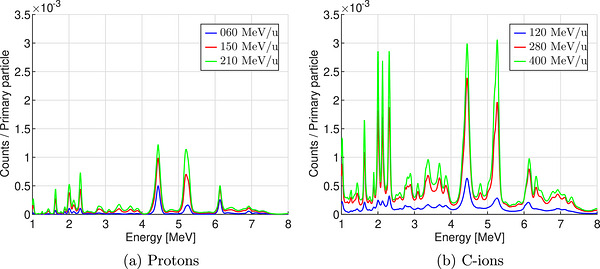
Primary gamma energy spectra for different proton and carbon‐ion energies.

The resulting spectra from the primary beam interaction are compared for each particle type in Figure 3. The results are normalized to a primary proton or ion. The analysis was restricted to a 1–8 MeV window, since this interval is the most relevant for prompt gamma imaging. Above 8 MeV, the intensity of emitted gamma rays decreases significantly. Since the analysis is limited only to primary gamma, all the prompt gamma considered are originated from the de‐excitation of nuclei undergoing a nuclear inelastic collision. Therefore, the energy spectra exhibit peaks corresponding to the excited states of the nuclei present in the tissue‐equivalent material. Table 3 lists emission lines of the most abundant nuclei present in the phantom, providing an explanation for the main peaks observed in the energy spectra.

**TABLE 3:** Main prompt gamma‐ray energies observed in the spectra of Figure 3, associated with the de‐excitation of excited nuclei produced in beam–target interactions.^20^, ^21^

$E_{\gamma }$ [MeV]	Reaction
1.0	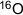 → 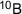 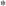
1.6	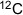 → 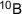 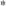 ; 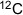 → 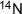 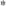
2.0	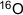 → 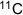 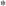 ; 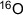 → 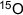 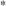
2.1	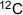 → 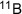 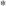
2.3	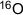 → 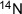 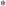
2.7	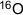 → 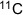 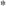 ; 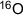 → 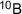 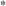 ; 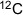 → 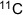 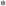 ; 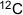 → 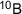 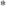
4.4	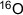 → 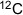 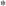 ; 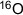 → 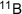 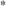 ; 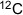 → 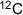 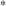 ; 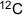 → 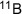 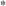
5.2	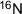 → 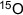 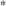 ; 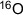 → 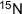 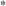
6.1	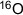 → 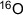 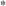 ; 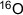 → 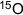 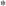

Compared to protons, carbon‐ions exhibit a higher yield for gamma production near 2 MeV, in particular for the peak at 2.1 MeV. The most prominent peaks appear at 4.4 MeV, 5.2 MeV, and 6.1 MeV, corresponding to gamma lines derived from various nuclear reactions with ^12^C, ^14^N, and ^16^O present in the phantom. In particular, the peaks correspond to the de‐excitation to the ground state of 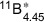, 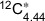, 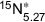, 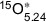, 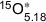, 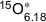, and 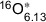.^1^

**FIGURE 4:**
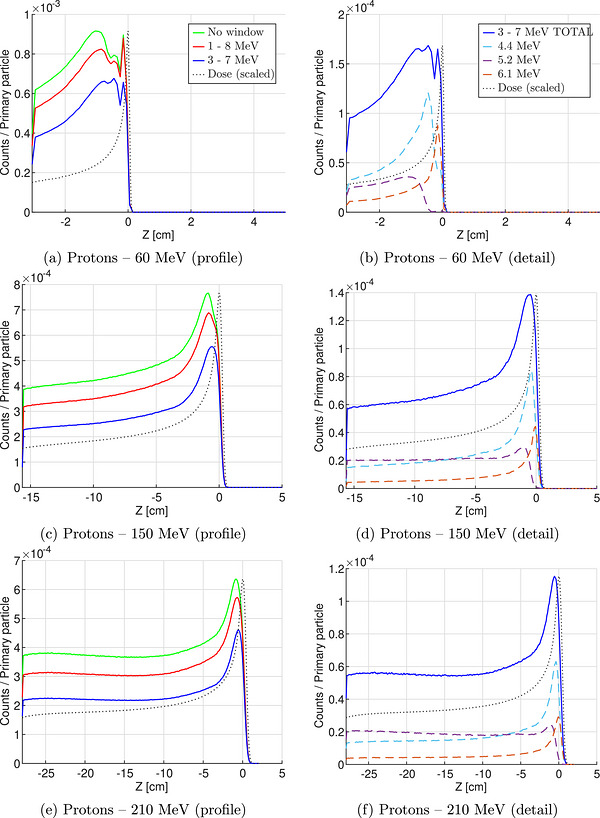
Gamma profiles and detailed distributions within the phantom for protons at various energies. The legend shown in (a) and (b) applies to all figures.

**FIGURE 5:**
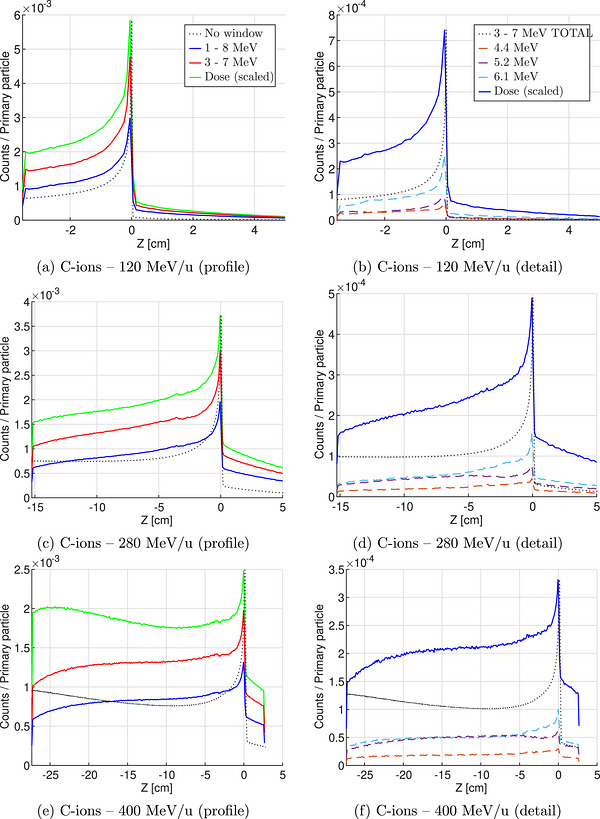
Gamma profiles and detailed distributions within the phantom for carbon‐ions at various energies. The legend shown in (a) and (b) applies to all figures.

Figures 4 and 5 illustrate the depth profiles of primary gammas for different energy selections. The left panels show, for each simulation set, the primary gammas filtered into three intervals: no energy window, 1–8 MeV, and 3–7 MeV. The 3–7 MeV energy window was introduced to investigate a more realistic operating condition for a gamma detection system, characterized by reduced counting rates, while preserving the main prompt gamma features relevant for range verification. In addition, photons below approximately 3 MeV include a significant contribution from neutron‐induced processes and scattered radiation, which are weakly correlated with the Bragg peak. Restricting the analysis to the 3–7 MeV interval therefore improves the signal‐to‐background ratio by suppressing low‐energy neutron‐induced gamma contributions, as also discussed in literature by *Zarifi et al.* ^35^ For clarity, the dose profile is scaled to match the maximum of the total profile. The fall‐off of the profiles of prompt gamma emission shows a clear correlation with the dose distribution and thus with the Bragg peak. The profiles for carbon‐ions appear narrower and with a sharper fall‐off than those for protons, which show a broader distribution. In the case of carbon‐ions, a tail beyond the Bragg peak is visible both in the dose distribution and the prompt gamma emission.

On the right side of Figures 4 and 5 the 3–7 MeV interval is further subdivided into smaller intervals corresponding to the main peaks observed in the energy spectra. This enables a more detailed analysis of the spatial profiles associated with specific nuclear de‐excitation lines. When energy filtering is applied, the fall‐off distribution changes shape due to the different thresholds of the reactions involved in prompt gamma generation.

Table 4 provides the yields per primary particle of photons and neutrons. The values for prompt gamma are reported for three different energy windows: no selection, 1–8 MeV, and 3–7 MeV.

**TABLE 4:** Production yield (counts per primary particle) for different projectiles and energies.

	Primary gamma			
**Beam**	No window	1–8 MeV	3–7 MeV	Neutrons
Proton 60 MeV	0.0236	0.0211	0.0117	0.00877
Proton 150 MeV	0.0759	0.0645	0.0374	0.07939
Proton 210 MeV	0.1132	0.0950	0.0558	0.15293
Carbon 120 MeV/u	0.1084	0.0825	0.0448	0.33178
Carbon 280 MeV/u	0.3762	0.2884	0.1508	2.16711
Carbon 400 MeV/u	0.5431	0.3809	0.1980	3.78692

*Note*: Values are reported for three energy windows of the emitted gammas, and for neutrons. The simulation statistical uncertainty is within 5% for all the reported values.

### 3.2 Neutron field

**FIGURE 6:**
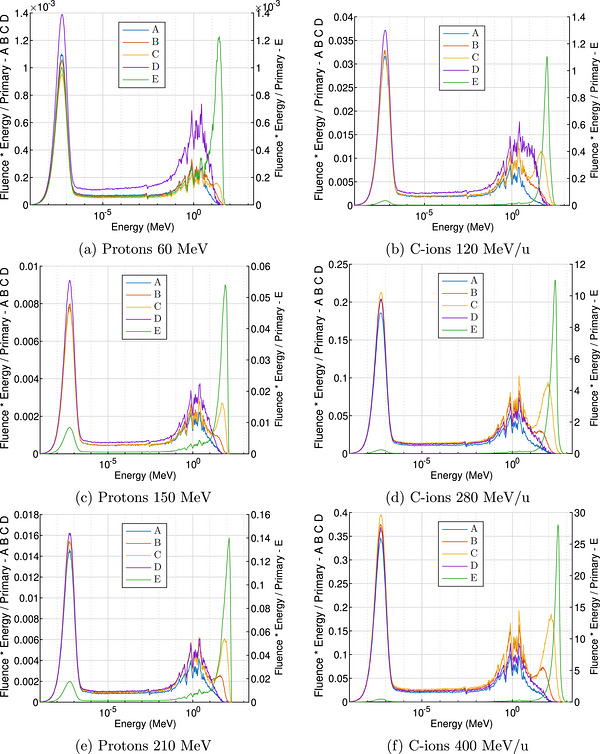
Neutron spectra scored in various locations around the room (see Figure 2). Due to the significantly higher fluence measured at location E, this spectrum is shown with a separate scale reported on the right.

For each simulation set, the neutron spectra collected in various locations of the room are plotted in Figure 6. For visualization purposes, the spectrum corresponding to position E is scaled differently, with its corresponding scale displayed on the right side of the plot. This is because this specific scoring volume is positioned directly in front of the phantom, facing the incoming beam. Due to the strong forward‐peaked distribution of fast neutrons, the fluence rate in this location is significantly higher, making it difficult to visualize the spectra in other regions of the room on the same scale.

The neutron spectra reveal the presence of thermal (<0.5 eV), epithermal (0.5 eV–0.1 MeV), and fast (>0.1 MeV) components. The spectra extend to higher energies with increasing beam energy, and the maximum neutron energies are higher for carbon‐ions compared to protons. While the neutron component below 10–20 MeV remains relatively constant throughout the room, the fluence of neutrons at higher energies is strongly dependent on the scoring position. Above this energy threshold, the spectra recorded at positions B, C, and E exhibit additional peaks correlated to the intranuclear cascade, with fluence increasing as the position approaches the beam axis in the distal region.

### 3.3 Detector signals

**FIGURE 7:**
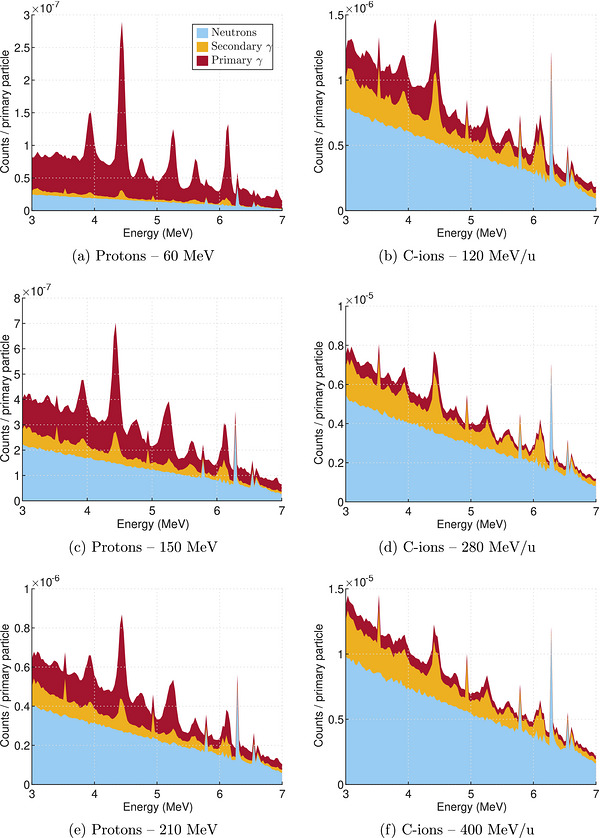
Energy spectra detected in the crystal region for different proton and carbon‐ion energies.The legend shown in (a) applies to all figures.

The detector signals for each simulation, subdivided into the radiation field components, are presented in Figure 7. The plots are shown within the 3–7 MeV energy window to better visualize the data and focus on the most relevant energy region. The signal component due to neutrons increases with beam energy and becomes dominant in the carbon‐ion cases. Additional peaks due only to neutron interactions with the detector are visible at 5.8 MeV, 6.3 MeV, 6.5 MeV and 6.6 MeV.

#### 3.3.1 1‐D profiles

**FIGURE 8:**
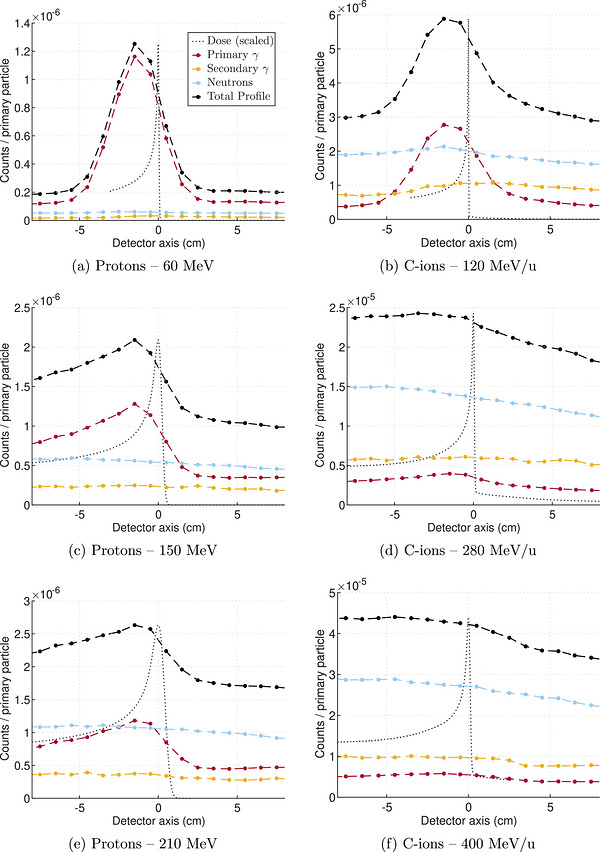
1‐D profiles of the detected signal in the crystal region for different proton and carbon‐ion energies (3–7 MeV energy window). The legend shown in (a) applies to all figures.

Figure 8 presents the resulting 1‐D profile obtained by including only prompt gammas in the 3–7 MeV energy window. The fall‐off related to the Bragg peak is more clearly visible in proton profiles than in carbon‐ion profiles, especially at lower energies. For carbon‐ions, the total profile exhibits a less pronounced fall‐off and a higher contribution from secondary components. The neutron and secondary gamma components form a relatively flat background. While secondary gammas are almost uniformly distributed, the neutron component shows an increase in the proximal region in respect to the beam.

**FIGURE 9:**
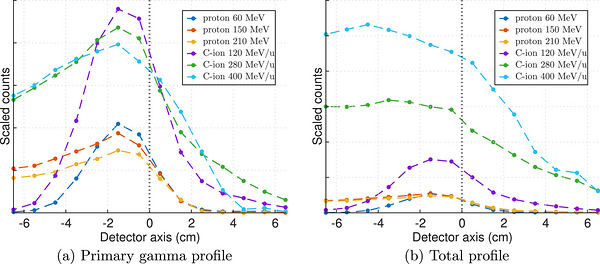
Primary gamma and total profiles detected in the crystal region.

The profiles vary across different orders of magnitude, particularly due to the different yields of the radiation field components in the case of carbon‐ion beams. Therefore, Figure 9 presents the six profiles of primary gamma radiation (Figure 9a) and the six profiles of the total detected signal (Figure 9b) scaled for better comparison. As the absolute quantities have already been shown in the previous figures, the profiles in Figure 9 are displayed after subtracting their minimum value, aligning all baselines to zero and thereby facilitating shape comparison. These plots are useful to highlight the slope induced by the BP, which would otherwise be less evident because of the large differences in component yields.

**FIGURE 10:**
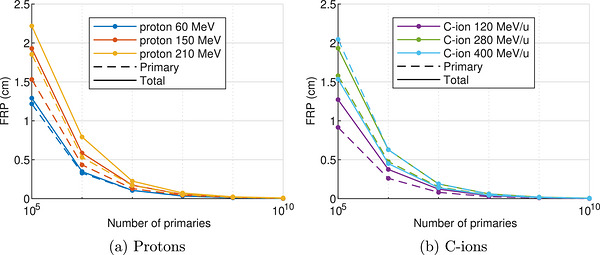
Fall‐off retrieval precision (FRP) as a function of the number of primary particles at different energies.

To quantify the statistical precision in retrieving the distal fall‐off position, the fall‐off retrieval precision (FRP) was evaluated as a function of the number of primary particles for the different beam energies. The results are shown in Figure 10 for proton and carbon‐ion beams.

In all cases, the FRP decreases with increasing number of primary particles, reflecting the improvement in statistical precision with increasing counting statistics. For proton beams, the FRP shown in Figure 10a is systematically lower for primary gammas than for the total detected profile. In addition, the FRP tends to increase with beam energy, indicating a reduced precision in determining the fall‐off position at higher proton energies.

A similar trend is observed for carbon‐ion beams, as shown in Figure 10b, where the FRP decreases with increasing number of primaries and generally increases with beam energy. However, for the highest energy configuration (400 MeV/u), the FRP obtained from the total signal is smaller than that obtained from primary gammas. The origin of this behavior is discussed in the following section.

## 4 DISCUSSION

The results of the Monte Carlo simulations provide different insights into the behavior of prompt and secondary radiation generated during hadrontherapy treatments, as well as their effects on a system based on a LYSO crystal.

### 4.1 Energy spectra and spatial profiles

The energy spectra of the primary gammas (Figure 3) exhibit distinct peaks corresponding to the de‐excitation process of the nuclei present in the tissue‐equivalent material. The most relevant lines for prompt gamma imaging are the 4.4 MeV line from ^12^C, the 5.2 MeV line from ^15^O, and the 6.1 MeV line from ^15^O and ^16^O.

Comparing the two particle types, carbon‐ions produce a higher prompt gamma yield than protons. This is primarily because carbon‐ions are heavier and more highly charged, increasing their probability to induce nuclear inelastic reactions along their path. Additionally, carbon‐ion spectra show extra peaks around 2 MeV due to prompt gamma emissions due to nuclear fragments. Indeed, the emission lines at approximately 2.0, 2.1, and 2.3 MeV correspond to ^11^C, ^11^B, and ^15^O, respectively. In particular, a large increase is observed for the 2.1 MeV line associated with ^11^B compared to the proton spectra.

After the energy spectra were analyzed, the spatial distribution of prompt gammas was investigated. Profiles presented in Figures 4 and 5 highlight the close correlation between prompt gamma emission and dose deposition for protons and carbon‐ions, respectively. As expected, the prompt gamma profile follows the shape of the dose distribution along the beam path and presents a fall‐off corresponding to the BP, confirming its suitability for range verification. While the overall shape of the profiles is comparable, some differences between protons and carbon‐ions can be noticed. In particular, the prompt gamma profile is broader for protons than for carbon‐ions, due to their lower mass and lower LET. Furthermore, while the proton profile ends after the fall‐off, the carbon‐ions profile exhibits a tail beyond the BP due to prompt gamma induced by nuclear fragments.

The filtered profiles in Figures 4, 5 (left) are essential to select the prompt gamma energy range with the highest yield, while optimizing the signal‐to‐background ratio. Here, energy filtering refers to the application of energy thresholds or energy windows at the data acquisition or analysis level. The results show that applying energy filtering does not compromise the spatial correlation with the BP: for all three energy windows, indeed, the fall‐off remains unchanged within the spatial and statistical resolution of the analysis. Notably, for the 400 MeV/u beam, energy filtering improves the profile by reducing the contribution of low‐energy photons in the initial region of the phantom. The main drawback is the reduction in absolute yield, which, however, can be acceptable in order to improve the acquisition system.

To quantify the stability of the fall‐off position, the depth at which the prompt gamma profile decreases to 50% of its maximum value ($z_{50}$) was extracted from the profiles obtained for the unfiltered spectrum and for the different energy windows. The differences between the corresponding $z_{50}$ values were found to be below 0.4 mm for all beam configurations. This value is smaller than the adopted spatial binning (1 mm) and therefore within the resolution of the present analysis. Consequently, the position of the fall‐off can be considered unchanged when applying the different energy selections.

The production yields reported in Table 4 quantitatively confirm the qualitative trends observed in the spectral and depth profile analyses. For completeness, neutron yields are also reported. For proton beams, the neutron yield per primary particle is of the same order of magnitude as the prompt gamma yield. In contrast, for carbon‐ions the neutron production becomes dominant. In particular, for the 400 MeV/u beam, the neutron yield per primary particle is approximately seven times higher than the corresponding prompt gamma yield. This marked increase in the neutron‐to‐prompt gamma ratio highlights the progressively more unfavorable signal‐to‐background conditions in carbon‐ion therapy and underlines the importance of properly accounting for neutron‐induced contributions in range monitoring systems.

Possible mitigation strategies include the use of neutron absorbers, such as highly borated materials, to reduce the thermal and epithermal neutron field, which is expected to contribute significantly to the detected background signal through neutron capture processes in the detector materials. Preliminary observations suggest that thermal neutrons play a relevant role and this aspect will be investigated more systematically in future studies. In addition, discrimination techniques based on pulse‐shape analysis (PSD) may allow the separation of neutron‐induced signals from prompt gamma interactions when using adequate scintillation crystals.

In addition to the considerations discussed above, the choice of the 3–7 MeV energy window is further supported by the presence of a high neutron field in the treatment room. Indeed, this window excludes lower‐energy gammas that are more likely to originate from scattering and secondary processes occurring within the room walls and surrounding materials. Specifically, this energy window avoids the 2.2 MeV gamma line produced by neutron capture on hydrogen, which contributes significantly to the counts but does not provide any spatial correlation with the BP.

A quantitative evaluation confirms this behavior. In the 0–3 MeV interval, secondary gammas account for approximately 14% –28% of the total detected signal depending on the beam configuration. This fraction decreases to about 4–8% in the 1–3 MeV region and further drops to approximately 1–3% in the 3–7 MeV window. Therefore, selecting the 3–7 MeV interval significantly suppresses the contribution of secondary gammas while preserving the prompt gamma signal correlated with the Bragg peak.

### 4.2 Neutron field

As previously mentioned, the analysis of the neutron field is crucial when considering the implementation of a detection system inside a treatment room. The energy spectra of the neutron field, shown in Figure 6, show the presence of thermal, epithermal, and fast neutron components. Thermal and epithermal neutrons are typically present in all neutron spectra, as they are produced through moderation processes in the surrounding materials, especially the treatment room walls. In contrast, fast neutrons, particularly those with energies above 10–20 MeV, are characteristic of interactions induced by high‐energy particle beams. These neutrons are produced from intranuclear cascade reactions, which are triggered when the projectile interacts inelastically with the nuclei in the target material. The spectrum of high‐energy neutrons is directly related to the primary beam, particularly to its energy per nucleon. As an example, for the most energetic beam simulated, the 400 MeV/u carbon‐ion beam, the neutron spectrum extends up to nearly 1 GeV.

As expected from the yields listed in Table 4, the neutron fluence in the energy spectra increases with the energy of the beam and is significantly higher for carbon‐ions than for protons. In fact, for the same penetration depth, carbon‐ions produce at least one order of magnitude more neutrons than protons. This difference is related to the higher nuclear cross‐section and fragmentation probability.

However, while the thermal and epithermal neutron components remain relatively uniform across the room, as shown in Figure 6, the fluence of fast neutrons varies considerably depending on the scoring position. Neutrons with energies above 10–20 MeV are strongly forward‐peaked along the beam direction, and this effect is clearly visible at positions B, C, and E, which show progressively increasing fluence. As shown in Figure 2, positions A and D are behind the fast neutron emission direction and thus record limited high‐energy neutron fluence.

This analysis underlines the importance of characterizing the various components of the radiation field. Understanding the energy composition of the neutron field is crucial, particularly when evaluating the signal‐to‐background ratio of detectors. Knowledge of the neutron energy spectrum allows for the design of optimized shielding to reduce the background and improve prompt gamma detection. Although the very fast component of the neutron field remains challenging to suppress due to its high energy and penetration power, significant improvements could be achieved by specifically addressing the thermal and epithermal components, which still represent a high fraction of the total field.

### 4.3 Detector signal

Figure 7 provides the analysis of the signal recorded in the scintillator detector, separated into its main components, offering a valuable insight into the influence of the radiation field in the treatment room. The neutron contribution increases with beam energy, as expected.

Consequently, secondary gammas, mainly produced by neutron interactions, increase along with them, although not at the same rate. In fact, neutron‐induced gammas are often emitted at lower energies, most notably around 2.2 MeV, outside the 3–7 MeV window considered. Additionally, secondary gammas that reach the detector after scattering within the room typically undergo further energy degradation. Moreover, the secondary gammas detected within this energy window exhibit the same spectral peaks as the primary prompt gammas discussed in Section 3.1, since high‐energy neutrons can trigger the intranuclear cascade as the primary particles.

On the other hand, neutrons mainly produce a continuous signal in the detector due to various scattering processes. However, additional peaks exclusively due to neutrons are visible above 5 MeV (i.e., 5.8, 6.3, 6.5, 6.6 MeV). These peaks result from gamma emissions following neutron capture on lutetium, one of the components of the detector material. Lutetium consists primarily of two isotopes: ^175^Lu, with a natural abundance of 97.4% and a neutron capture cross section of 23 barns, and ^176^Lu, with 2.6% abundance and a significantly higher cross section of 2090 barns.

Even if the energy deposited comes from gamma rays emitted during the capture process, the signal is attributed to neutrons in the signal plots, as the reaction takes place inside the detector itself. As previously noted, the signal associated only to neutrons in Figure 7 can therefore be largely attributed to the thermal component, as suggested by these distinct peaks, which are solely associated with neutron capture events inside the detector.

The realistic energy resolution of the scintillator (i.e., 10%) was not applied in order to preserve the visibility of narrow spectral structures (e.g., gamma lines from neutron capture in lutetium) that would otherwise merge with broader prompt gamma features.

To study the spatial distribution of the detected signal, the profiles detected are presented in Figure 8. This comparison highlights that the slope related to the BP is more noticeable in the profiles associated to proton beams than to carbon‐ions, and it is more evident for lower energies. This is consistent with all the observations made in the previous sections on the relation and distribution of the various components.

For carbon‐ions, the higher initial dose and extended tail due to fragmentation reduce the contrast between the BP and the non‐relevant regions of the profile, making the fall‐off less pronounced with respect to protons. Moreover, the tail resulting from nuclear fragmentation causes a forward shift of the 1D profile, as shown in Figure 9a. Furthermore, the neutron and secondary gamma components contribute a relatively uniform background that further flattens the signal. This behavior is consistent with the ratio between neutron and primary gamma yields reported in Table 4. The higher neutron production leads to a flatter profile, making the fall‐off slope expected at the BP less evident. A small difference can be noted between the neutron and secondary gamma contributions to the profile: while secondary gammas tend to form a nearly uniform background, the neutron component shows a slight increase in the entrance region of the phantom, likely due to the fact that neutrons are primarily produced before the BP, where the beam energy is higher. These differences in the shape of the detected profiles also affect the statistical precision with which the fall‐off position can be determined. To quantify this effect, the fall‐off retrieval precision (FRP) was evaluated as a function of the number of primary particles.

The comparison between primary gammas and the total detected profiles highlights the impact of secondary radiation on the precision of the fall‐off determination. For proton beams, as shown in Figure 10a, the FRP obtained using only primary gammas is systematically lower than that obtained from the total detected signal. This behavior is expected, since the total signal includes contributions from neutrons and secondary gamma radiation, which introduce additional fluctuations that are not directly correlated with the Bragg peak position.

The FRP also shows a dependence on beam energy. In particular, the precision tends to degrade with increasing proton energy. This trend can be related to the broader spatial distribution of prompt gamma emission at higher energies, which results in a less steep distal fall‐off of the detected profiles, as previously observed in Figure 8. As the fall‐off becomes less pronounced, the determination of its position becomes more sensitive to statistical fluctuations in the detected signal.For carbon‐ion beams, a similar trend is observed (Figure 10b), where the FRP decreases with increasing number of primaries and generally increases with beam energy.

It should be noted that the FRP values obtained for proton and carbon‐ion beams are of the same order of magnitude when evaluated for a fixed number of primary particles. However, this comparison does not correspond to equivalent clinical irradiation conditions. Due to the higher relative biological effectiveness of carbon ions and to the dependence of energy loss on $1/Z^{2}$, resulting in a higher stopping power and a higher energy deposition, a smaller number of primary particles is required to deliver the same dose to the patient. Consequently, the number of detected prompt gammas per treatment spot is expected to be lower in carbon‐ion therapy. In typical clinical conditions, the number of protons per spot can reach approximately $10^{8}$–$10^{9}$, whereas the number of carbon ions is usually of the order of $10^{6}$–$10^{7}$. As a consequence, when comparing the FRP at clinically relevant intensities, proton beams achieve sub‐millimetric precision (approximately 0.2–0.7 mm), while carbon‐ion beams exhibit a reduced precision, typically in the millimetric range (approximately 2–6 mm), due to the lower number of detected prompt gammas per spot. Therefore, although the FRP obtained in the simulations is comparable for the same number of primaries, the achievable statistical precision in realistic treatment conditions may be more challenging for carbon‐ion beams due to the reduced number of incident particles.

A comparison between the lowest‐energy configurations further highlights the role of secondary radiation. In the case of the 60 MeV proton beam, the FRP obtained from primary and total signals is almost identical, indicating a negligible contribution from secondary components. In contrast, for the 120 MeV/u carbon‐ion beam the difference between the primary and total FRP is significantly larger, reflecting the stronger influence of neutron‐induced and secondary gamma radiation on the detected profiles. However, for the highest energy configuration (400 MeV/u), the FRP obtained from the total signal is slightly smaller than that obtained using only primary gammas. This effect is attributed to the geometrical limitation of the phantom used in the simulations. In this configuration, the Bragg peak is located close to the distal edge of the phantom, so that particles and nuclear fragments traveling beyond this region are not fully contained within the phantom volume. As a consequence, a fraction of forward‐going radiation, including fragments, neutrons, and the corresponding secondary gammas, is lost. This effect artificially sharpens the distal fall‐off of the total detected profile, producing an apparently improved FRP that does not correspond to a real improvement in the range sensitivity. It should be noted that this effect would be present also in a real patient depending on the irradiation geometry.

It should be underlined that this study adopts a simplified geometry to isolate the physical drivers of prompt‐gamma and neutron contributions. In fact, the phantom is homogeneous soft tissue and does not represent patient heterogeneities. Moreover, the irradiation conditions employ mono‐energetic pencil beams and do not model full treatment plans. Finally, an intrinsic scintillator energy resolution was not applied to preserve narrow spectral structures for analyzing its main components; in clinical detectors, these features may be broadened and partially merge with prompt‐gamma lines, although the underlying neutron‐capture mechanism remains relevant for background formation.

Nevertheless, the component detector analysis provides practical guidance for any future PGI system optimization. In particular, especially for carbon‐ion beams, restricting the analysis to the 3–7 MeV window improves signal‐to‐background conditions by suppressing neutron‐induced contributions while preserving the Bragg‐peak‐correlated prompt‐gamma component. However, the strong neutron field and neutron‐capture processes in the scintillation crystal introduce additional background and flatten the range‐sensitive fall‐off, emphasizing the need for moderated‐neutron mitigation and Monte Carlo informed interpretation strategies in carbon‐ion PGI. It should be noted that, all the analysis performed in this work can be extended to any scintillation crystal.

## 5 CONCLUSION

This work presented a Monte Carlo investigation of prompt gamma emission and neutron background in proton and carbon‐ion therapy, performed using the FLUKA code and a simulated prompt gamma imaging system based on a knife‐edge collimator coupled to a LYSO scintillator detector. The simulations aimed at performing a systematic comparison between proton and carbon‐ion beams at clinically relevant energies. The results confirm the spatial correlation between prompt gamma emission and the Bragg peak for both particle types, supporting PGI for range verification. However, carbon‐ion beams produce a stronger neutron background, which increases with energy and reduces the contrast of the distal fall‐off. The analysis of the simulated detector supports the choice of the 3–7 MeV energy window, where secondary gamma contributions are strongly suppressed while preserving the Bragg‐peak‐correlated prompt gamma signal, thus improving the signal‐to‐background conditions.

The statistical precision in retrieving the distal fall‐off position was evaluated through FRP as a function of the number of primary particles. The comparison between primary and total detected profiles shows that secondary radiation, mainly neutrons and secondary gammas from, for example, the concrete walls, degrades the precision of the fall‐off determination by introducing fluctuations not correlated with the Bragg peak. Overall, these results provide a characterization of the radiation components affecting prompt gamma detection in hadrontherapy and highlight the main physical mechanisms that influence the accuracy of range verification. These findings provide guidance for the design and optimization of prompt gamma detection systems for real‐time range monitoring, especially in the more challenging radiation environment associated with carbon‐ion therapy.

## CONFLICT OF INTEREST STATEMENT

The authors declare no conflicts of interest.

## Footnotes

1 The notation  indicates an excited nucleus of mass number A and chemical symbol X, at an excitation energy E in MeV.
